# Identification of chemogenomic features from drug–target interaction networks using interpretable classifiers

**DOI:** 10.1093/bioinformatics/bts412

**Published:** 2012-09-03

**Authors:** Yasuo Tabei, Edouard Pauwels, Véronique Stoven, Kazuhiro Takemoto, Yoshihiro Yamanishi

**Affiliations:** ^1^ERATO Minato Project, Japan Science and Technology Agency, Sapporo 060-0814, Japan; ^2^Mines ParisTech, Centre for Computational Biology, 35 rue Saint-Honore, F-77305 Fontainebleau Cedex, France; ^3^Institut Curie, F-75248 Paris, France; ^4^INSERM U900, F-75248 Paris, France; ^5^Department of Bioscience and Bioinformatics, Kyushu Institute of Technology, Kawazu 680-4, Iizuka, Fukuoka 820-8502, Japan; ^6^ PRESTO, Japan Science and Technology Agency, Kawaguchi, Saitama 332-0012, Japan; ^7^Division of System Cohort, Medical Institute of Bioregulation, Kyushu University, 3-1-1 Maidashi, Higashi-ku, Fukuoka, Fukuoka 812-8582, Japan

## Abstract

**Motivation:** Drug effects are mainly caused by the interactions between drug molecules and their target proteins including primary targets and off-targets. Identification of the molecular mechanisms behind overall drug–target interactions is crucial in the drug design process.

**Results:** We develop a classifier-based approach to identify chemogenomic features (the underlying associations between drug chemical substructures and protein domains) that are involved in drug–target interaction networks. We propose a novel algorithm for extracting informative chemogenomic features by using *L*_1_ regularized classifiers over the tensor product space of possible drug–target pairs. It is shown that the proposed method can extract a very limited number of chemogenomic features without loosing the performance of predicting drug–target interactions and the extracted features are biologically meaningful. The extracted substructure–domain association network enables us to suggest ligand chemical fragments specific for each protein domain and ligand core substructures important for a wide range of protein families.

**Availability:** Softwares are available at the supplemental website.

**Contact:** yamanishi@bioreg.kyushu-u.ac.jp

**Supplementary Information:** Datasets and all results are available at http://cbio.ensmp.fr/~yyamanishi/l1binary/ .

## 1 INTRODUCTION

Drug phenotypic effects are caused by the interactions between drug molecules and their target proteins including their primary targets and off-targets (Blagg, 2006; Whitebread *et al.*, 2005). Polypharmacology, the idea that drug phenotypic effects are not due only to its primary target, but rather to its whole spectrum of interactions, tends to become a new paradigm in drug design. It is important to identify the molecular mechanisms behind overall drug–target interactions or more generally compound–protein interactions, leading to many applications at different levels of the drug design process. There is a hypothesis that polypharmacology is strongly involved in both drug chemical substructures and protein functional sites, so there is a strong incentive to develop new methods to explore the association between drug chemical substructures and protein functional sites in terms of drug–target interactions.

Docking (Kolb *et al.*, 2009) or ligand–based approach (e.g. QSAR) have been proposed to analyze and predict interactions with respect to a single protein, so these methods cannot be applied to mine ligand–protein pairs across many different proteins. Chemogenomics is an emerging research area that attempts to associate the chemical space of possible ligands with the genomic space of possible proteins (Dobson, 2004; Kanehisa *et al.*, 2006; Stockwell, 2000). Following this principle, several statistical methods have been proposed to predict drug–target or ligand–protein interactions on a large scale. (Faulon *et al.*, 2008; Jacob and Vert, 2008; Keiser *et al.*, 2009; Li *et al.*, 2011; Yabuuchi *et al.*, 2011; Yamanishi *et al.*, 2008; Yang *et al.*, 2009). These methods are purely predictive and do not provide any further understanding of molecular mechanisms behind ligand–protein interactions. Drug–target interactions are due to drug chemical substructures and protein functional sites. Beyond the ligand–protein interaction prediction problem, a variety of methods have been proposed to investigate the correlation between chemical substructures, biological activities and phenotypic effects (Han *et al.*, 2008; Klekota and Roth, 2008; Pauwels *et al.*, 2011; Shigemizu *et al.*, 2009). Several methods based on binding pockets comparison have been proposed (Hoffmann *et al.*, 2010; Morris *et al.*, 2005; Najmanovich *et al.*, 2008), but they require the knowledge of protein 3D structures, which is not genome-wide available. However, most previous works have been performed from the viewpoint of either chemical substructures or protein functional sites.

One of the most challenging issues in recent chemogenomic research is to identify the underlying associations between drug chemical substructures and protein functional sites which are involved in drug–target interaction networks. Recently, a variant of sparse canonical correspondence analysis (SCCA) has been proposed to extract sets of chemical substructures and protein domains governing drug–target interactions (Yamanishi *et al.*, 2011), but the variation of detectable protein domains is very limited. The use of both graph mining and sequence mining has been proposed to extract drug substructures and protein subsequences which tend to appear in known drug–target interactions (Takigawa *et al.*, 2011). However, the size of extracted subsequences is very small (e.g. two or three amino acids), which makes biological interpretation difficult, and any prediction framework for new interactions based on the extracted features was not provided.

In this article, we develop a classifier-based approach to identify chemogenomic features (the underlying associations between drug chemical substructures and protein domains) which are strongly involved in drug–target interaction networks. We propose a novel algorithm for extracting informative chemogenomic features by using *L*_1_ regularized classifiers over the tensor product space of possible drug–target pairs. In the results, we show that the proposed method can extract a very limited number of chemogenomic features without loosing the performance of predicting drug–target interactions. We underline that the extracted chemogenomic features are biologically meaningful and discuss how the method can help the drug development process. The extracted substructure–domain association network enables us to suggest ligand chemical fragments specific for each protein domain and ligand core substructures important for a wide range of protein families.

## 2 MATERIALS

Drug–target interactions involving human proteins were obtained from the DrugBank database (Wishart *et al.*, 2006). Target proteins belong to many different classes such as enzymes, ion channels, G protein-coupled receptors (GPCRs) or nuclear receptors. The dataset consists of 4809 drug–target interactions involving 1862 drugs and 1554 target proteins. The set of interactions is used as gold standard data. This is the same drug–target interaction data used in the previous study (Yamanishi *et al.*, 2011).

Chemical structures of drugs were encoded by a chemical fingerprint corresponding to 881 chemical substructures defined in the PubChem database (Chen *et al.*, 2009). Chemically identical drugs with the same structures (duplicates) are removed, so structures of all drugs in the above interaction data are unique. Each drug was represented by an 881 dimensional binary vector whose elements encode for the presence or absence of each PubChem substructure by 1 or 0, respectively. Among the 881 substructures used to represent the chemical structures, 663 are actually used, because 218 do not appear in our drug set.

Genomic information about target proteins was obtained from the UniProt database (Consortium, 2010), and associated protein domains were obtained from the PFAM database (Finn *et al.*, 2008). Target proteins in our dataset were associated with 876 PFAM domains. Each target protein was represented by a 876 dimensional binary vector whose elements encode for the presence or absence of each of the retained PFAM domain by 1 or 0, respectively.

## 3 METHODS

We solve the typical *in silico* chemogenomics problem as the following machine learning problem: given a collection of *n* drug–target pairs (*C*_1_,*P*_1_),...,(*C_n_*,*P_n_*), known to interact or not, estimate a function *f* (*C,P*) that would predict whether a compound *C* interacts with a protein *P*. In addition, our task includes extracting features which effectively contribute to the prediction. In this section, we propose a general approach to solve these problems in a unified framework.

### 3.1 Model

Linear model is a useful tool for classification and regression. Generally, a linear model represents each example *E* by a feature vector representation Φ(*E*) ∈ ℜ^*D*^ and then estimates a linear function *f*(*E*) = ***w***^T^Φ(*E*) whose sign is used to predict whether the example *E* is classified into positive or negative. The weight vector ***w*** ∈ ℜ^*D*^ is estimated based on its ability to correctly predict the classes of examples in the training set. In addition to its classification ability, linear models have an interpretability of features. Since each element of a feature vector Φ(*E*) corresponds to an element of its weight vector ***w***, we can extract effective features contributing to the prediction by sorting elements of Φ(*E*) according to the values of the corresponding elements of ***w***.

The prediction of drug–target interactions or compound–protein interactions is more complicated because the dataset consist of drug–target pairs or compound–protein pairs. Let *C* be a drug (or drug candidate compound) and *P* be a target (or target candidate protein). To apply the previous machine learning approach to this problem, we need to represent a pair of a compound *C* and a protein *P* by a feature vector Φ(*C, P*) and then estimate a linear function *f*(*C, P*) = ***w***^T^ Φ(*C, P*) whose sign can be used to predict whether a pair of *C* and *P* interacts or not. The weight vector ***w*** is estimated based on its ability to correctly predict interactions of drug–target pairs or compound-protein pairs.

### 3.2 Vector representation of drug–target pairs

We propose to represent a compound–protein pair by a feature vector using the tensor product of two feature vectors. The representation is similar to the representations in (Jacob and Vert, 2008) and (Faulon *et al.*, 2008).

The fingerprint of a compound *C* is represented as *D*-dimensional binary vector: Φ(*C*) = (*c*_1_,*c*_2_,*...,c_D_*)^T^ where *c_i_* ∈ {0,1},*i* = 1,*...,D*. A fingerprint of a protein *P* is represented as *D*′-dimensional binary vector as well: Φ(*P*) = (*p*_1_,*p*_2_,*...,p_D_*′)^T^ where *p_i_* ∈ {0,1},*i* = 1,*...,D*′. We define a fingerprint of a drug–target pair as the tensor product of Φ(*C*) and Φ(*P*) as follows:

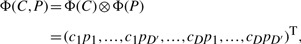

where Φ(*C, P*) consists of all possible products of elements in feature vectors Φ(*C*) and Φ(*P*), and thus is a *D*×*D*′ dimension binary vector. Since Φ(*C*) and Φ(*P*) encode for chemical substructures and protein domains, respectively, each element in Φ(*C, P*) corresponds to a pair of a chemical substructure and a protein domain.

### 3.3 Binary classifiers

We apply two popular binary linear classifiers: logistic regression and linear support vector machine (SVM). Models are typically learned to minimize objective functions with a regularization for both classifiers. It is well known that the use of regularization is necessary to achieve a model that generalizes well to unseen data, particularly if the dimension of features is very high relative to the amount of training data. One common regularization is *L*_2_-regularization which keeps most elements in the weight vector to be non-zeros. Therefore, one can suffer from interpreting features from learned weights. We shall, respectively, refer to *L*_2_-regularized logistic regression and linear SVM as L2LOG and L2SVM. Another possible regularization is *L*_1_-regularization that makes most elements in the weight vector to be zeros. In this study, we introduce logistic regression and linear SVM with *L*_1_-regularization for its high interpretability.

Given a set of drug–target pairs and labels (Φ(*C_i_*, *P_i_*),*y_i_*),*y_i_* ∈ {+1,–1}, logistic regression and linear SVM are, respectively, formulated by the following unconstrained optimization problems:
(1)
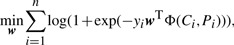

and
(2)
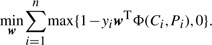

To enhance the interpretability of linear models, the weight vector is optimized with *L*_1_-regularization as follows:
(3)
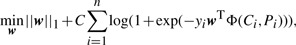

and
(4)
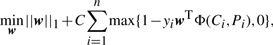

where ||·||_1_ is *L*_1_ norm (the sum of absolute values in the vector) and *C* is a hyper-parameter. *L*_1_-regularization has an effect that makes the weights of uninformative features zeros without loss of classification accuracy. We shall refer to *L*_1_-regularized logistic regression and linear SVM as L1LOG and L1SVM, respectively.

Learning weight vectors from high dimensional data is a difficult problem. For drug–target interaction predictions, the dimension of a feature vector Φ(*C, P*) tends to be very high. In our dataset, the dimension of Φ(*C, P*) is 663×876 = 584103. To overcome this difficulty, SVM with pairwise kernels was used in the previous works (Faulon *et al.*, 2008; Jacob and Vert, 2008), which is referred to as Kernel-SVM (KSVM). However, it is difficult to apply KSVM to large-scale interaction predictions. This is because the time complexity of the quadratic programming problem for the KSVM is *O*(*n_d_*^3^ × *n_t_*^3^), where *n_d_* is the number of drugs and *n_t_* is the number of target proteins, and even worse the space complexity is *O*(*n_d_*^2^× *n_t_*^2^), which is just for storing the pairwise kernel matrix. Moreover, KSVM does not have an interpretability of features.

A crucial observation is that Φ(*C,P*) is a sparse binary vector. For such sparse binary vectors, weight vectors can be learned via efficient optimization algorithms (Hsieh *et al.*, 2008). The software is available from http://www.csie.ntu.edu.tw/~cjlin/liblinear/.

## 4 RESULTS

### 4.1 Extraction of chemogenomic features

We tested the feature extraction ability of five feature extraction methods: L1LOG, L1SVM, L2LOG, L2SVM and SCCA. Note that L1LOG and L1SVM are the proposed methods with L1-regularization, L2LOG and L2SVM are the proposed methods with L2-regularization and SCCAis the previous method (Yamanishi *et al.*, 2011). We extracted chemogenomic features that were positively weighted in each method. The parameters in each method (e.g. regularization parameters, sparsity parameters and number of components) were optimized by performing cross-validation. (More details of the cross-validation procedure will be explained in the next subsection).

**Fig. 1.**
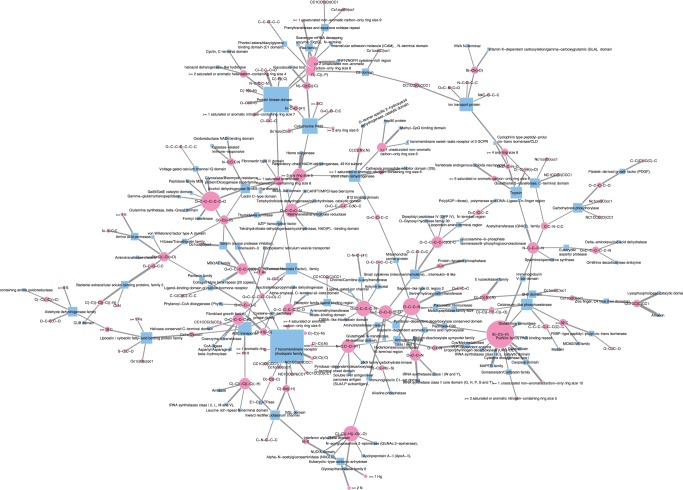
Part of the extracted substructure–domain association network. Pink circle and blue rectangle represent a chemical substructure and a protein domain, respectively. Node size represents a node degree. Edge width represents the weight of substructure and domain pair

Each chemogenomic feature consists of a chemical substructure and a protein domain which are suspected of being associated with each other in terms of drug–target interactions. We evaluated the strength of the association between chemical substructures and protein domains by the corresponding weight in the classifier. Figure 1 shows a global view of substructure–domain association network behind the drug–target interaction network. We focused on the maximal network component because of space limitation and on chemical substructures and protein domains whose weights were higher than 0.4 in the case of the L1SVM method for visualization simplicity in the figure. Table 1 shows examples of highly weighted chemogenomic features extracted by the L1LOG method. The result of all extracted features in each method can be obtained from the Supplementary Materials.

**Table 1.** Examples of extracted chemogenomic features by the L1LOG method

Rank	Weight	Substrucure ID Domain ID	PubChem substructure definition PFAM domain definition
1	2.1468	SUB158	>= 3 any ring size 5
1	2.1468	PF00106	short chain dehydrogenase
2	2.1118	SUB414	S(^~^C)(^~^H)
2	2.1118	PF00255	Glutathione peroxidase
3	1.9413	SUB158	>= 3 any ring size 5
3	1.9413	PF01126	Heme oxygenase
4	1.8035	SUB686	O = C-C-C-C-N
4	1.8035	PF01094	Receptor family ligand binding region
5	1.7707	SUB687	O = C-C-C-C-O
5	1.7707	PF03171	2OG-Fe(II), oxygenase superfamily
6	1.7514	SUB348	C(^~^C)(^~^H)(^~^O)(^~^O)
6	1.7514	PF03414	Glycosyltransferase family 6
7	1.6343	SUB387	C(:C)(:C)(:N)
7	1.6343	PF00042	Globin
8	1.6299	SUB409	O(^~^H)(^~^S)
8	1.6299	PF00167	Fibroblast growth factor
9	1.5807	SUB32	>= 2 P
9	1.5807	PF00348	Polyprenyl synthetase
10	1.5797	SUB567	O-C-C-N
10	1.5797	PF00464	Serine hydroxymethyltransferase
11	1.5105	SUB309	O-H
11	1.5105	PF00102	Protein-tyrosine phosphatase
12	1.5065	SUB433	C(-C)(-C)(= O)
12	1.5065	PF02518	Histidine kinase-, DNA gyrase B-, and HSP90-like ATPase
13	1.5033	SUB449	C(-H)(= O)
13	1.5033	PF00107	Zinc-binding dehydrogenase
14	1.4956	SUB695	O = C-C-C-C-C = O
14	1.4956	PF00551	Formyl transferase
15	1.4784	SUB433	C(-C)(-C)(= O)
15	1.4784	PF07884	Vitamin K epoxide reductase family

**Fig. 2.**
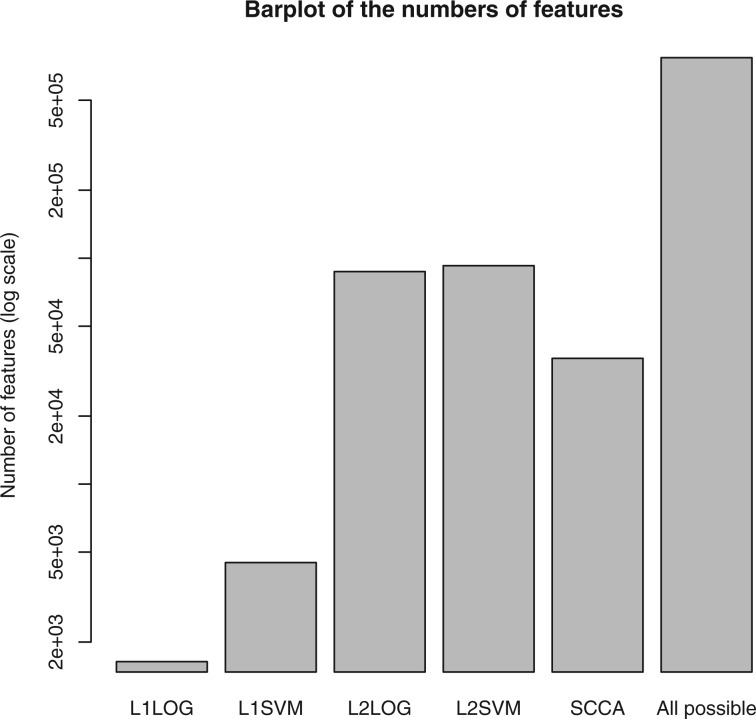
Comparison of the number of extracted features between different methods

Figure 2 shows a comparison of the number of extracted features between the five different feature extraction methods. In the case of SCCA, we evaluated the association between chemical substructures and protein domains by computing the product of their weight elements between chemical substructures and protein domains within each canonical component, and we took unique combinations as chemogenomic features if they were present in different canonical components. It was observed that L1LOG and L1SVM extracted a very limited number of features, compared with other methods, owing to the sparsity of the L1 penalty-based methods. This allows meaningful analysis of the extracted features for biological interpretation, which will be shown in the biological interpretation subsection.

**Fig. 3.**
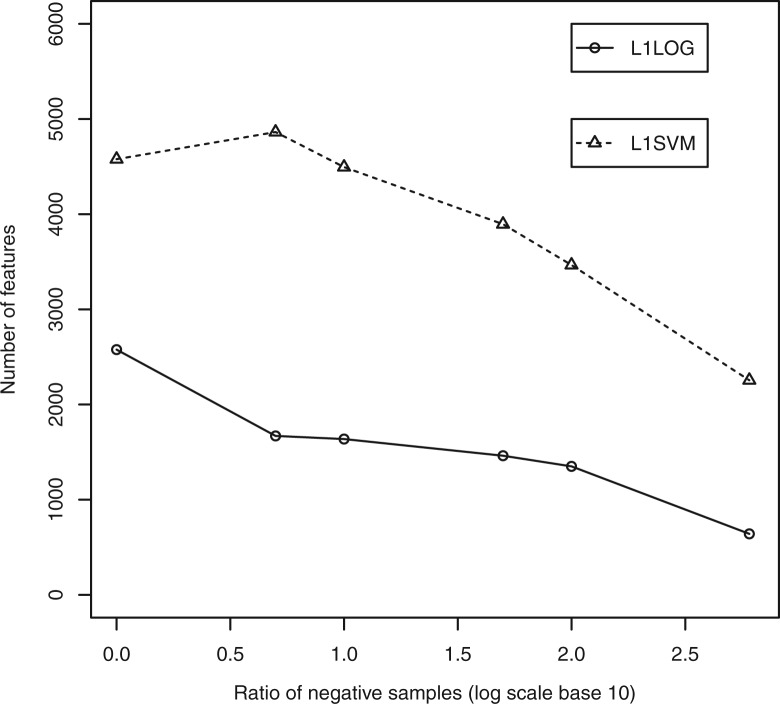
Effect of negative examples on the number of extracted features

We examined the effect of the ratio of negative samples on the number of extracted features in the case of L1LOG and L1SVM. Figure 3 shows the number of extracted features against the ratio of negative samples. We found a tendency that as the ratio of negative samples increases, the number of features decreases in both L1LOG and L1SVM.

**Fig. 4.**
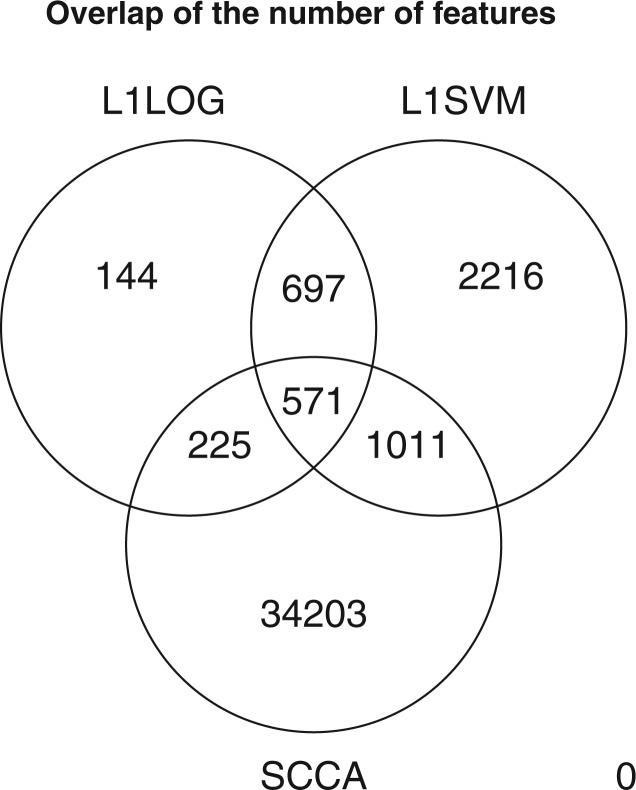
Distribution of the number of extracted features across different feature extraction methods

We also examined the distribution of extracted features between three feature extraction methods: L1LOG, L1SVM and SCCA, where the number of negative examples was 10 times larger than the number of positive examples. Figure 4 shows a Venn diagram of the number of unique chemogenomic features across L1LOG, L1SVM and SCCA. The numbers of extracted features by L1LOG and L1SVM are much fewer than that by SCCA. This result suggests that the L1-regularized classifier-approach enables us to reduce the number of features, compared with the previous method.

### 4.2 Performance evaluation

If the extracted chemogenomic features are biologically meaningful and capture relevant information with respect to protein–ligand interactions, one would expect that they present good generalization properties to reconstruct known drug–target interactions from the extracted features. We tested the prediction performance of L1LOG, L1SVM, L2LOG, L2SVM and SCCA. We also tested the prediction performance of Kernel SVM (KSVM) (Faulon *et al.*, 2008; Jacob and Vert, 2008). Note that KSVM is the state-of-the-art method for ligand–protein interaction prediction, but it cannot extract any information about important molecular features nor provide any biological interpretation since it only predicts interactions.

We performed two types of cross-validations: pair-wise cross-validation and block-wise cross-validation. In the pair-wise cross-validation we performed the following 5-fold cross-validation. (i) We randomly split drug-target pairs in the gold standard set into five subsets of roughly equal sizes and took each subset in turn as a test set. (ii) We trained a predictive model on the remaining four subsets. (iii) We computed the prediction scores for drug–target pairs in the test set. (iv) Finally, we evaluated the prediction accuracy over the 5-folds. Pair-wise cross-validation assumes the situation where we want to detect missing interactions between known drugs (e.g. marketed drugs) and known target proteins (e.g. known therapeutic targets) with information about interaction partners. In the block-wise cross-validation we performed the following 5-fold cross-validation. (i) We randomly split drugs and target proteins in the gold standard set into five drug subsets and five target subsets, and took each drug subset and each target subset in turn as test sets. (ii) We trained a predictive model on drug–target pairs in the remaining four drug subsets and four target subsets. (iii) We computed the prediction scores for drug–target pairs involving test drug set and test target set. (iv) Finally, we evaluated the prediction accuracy over the 5-folds. Block-wise cross-validation assumes the situation where we want to predict unknown target proteins of newly coming drug candidate compounds (e.g. newly synthesized compounds) and unknown ligands of newly coming target candidate proteins (e.g. orphan proteins).

We evaluated the performance by the receiver operating characteristic (ROC) curve, which is a plot of true positives as a function of false positives based on various thresholds, where true positives are correctly predicted interactions and false positives are predicted interactions that are not present in the gold standard interactions. We summarized the performance by the area under the ROC curve (AUC) score, where 1 is for a perfect inference and 0.5 is for a random inference. We repeated the cross-validation experiment five times, and computed the average of the AUC scores over the five cross-validation folds. The parameters involved in the other methods were optimized with the AUC score as the objective function.

Table 2 shows the AUC scores by the pair-wise cross-validation, where the number of negative examples is varied from the same number of positive examples to the number of all possible negative examples. It was observed that all classifiers worked well when there were many negative examples. However, KSVM did not work with a large number of negative examples because of the problem of memory shortage and computational cost in the training process. It also seemed that the proposed methods L1SVM, L1LOG, L2SVM and L2LOG outperformed the previous methods KSVM and SCCA. SVM-based methods seemed to be better than LOG-based methods in terms of AUC. The prediction accuracies of *L*_1_-regularized methods was close to or slightly worse than that of *L*_2_ regularized methods. These results suggest that L1-regularized methods provide us with more selective drug substructures and protein domains, without losing important information encoding protein–ligand interactions.

**Table 2.** AUC scores on pair-wise cross validation experiments

Ratio	L_1_-Log	L_1_-SVM	L_2_-Log	L_2_-SVM	KSVM	SCCA
1	0.8285±0.0009	0.8301±0.0006	0.8366±0.0010	0.8461±0.0009	0.8339±0.0005	0.7975±0.0018
5	0.8379±0.0008	0.8551±0.0008	0.8464±0.0008	0.8659±0.0008	NA	0.7975±0.0018
10	0.8437±0.0010	0.8654±0.0010	0.8512±0.0010	0.8728±0.0009	NA	0.7975±0.0018
50	0.8419±0.0010	0.8677±0.0009	0.8514±0.0011	0.8736±0.0010	NA	0.7975±0.0018
100	0.8418±0.0010	0.8677±0.0009	0.8516±0.0010	0.8740±0.0010	NA	0.7975±0.0018
ALL	0.8411±0.0006	0.8658±0.0006	0.8483±0.0007	0.8659±0.0004	NA	0.7975±0.0018

The number of negative examples is varied from the same number of positive examples to the number of all possible negative examples. NA means that it was not computationally feasible.

Table 3 shows the AUC scores by the block-wise cross-validation, where the number of negative examples is varied from the same number of positive examples to the number of all possible negative examples. In this case, the same effects of negative examples were observed as those in the pair-wise cross-validation, but there was little significant difference in the prediction accuracy between different methods. The AUC scores in the block-wise cross-validation were lower than those in the pair-wise cross-validation, which implies that predicting unknown interactions for newly coming drug candidate compounds and target candidate proteins is much more difficult than detecting missing interactions between known drugs and known target proteins. In practice, we found it very difficult to analyze the extracted chemogenomic features when there were too many highly or lowly weighted elements like L2LOG and L2SVM. On the contrary, the advantage of L1-regularized classifiers over L2-regularized classifiers is the ability to derive biological interpretations, as shown on a few examples in the next subsection.

**Table 3.** AUC scores on block-wise cross validation experiments

Ratio	L_1_-Log	L_1_-SVM	L_2_-Log	L_2_-SVM	KSVM	SCCA
1	0.7071±0.0010	0.7061±0.0015	0.7222±0.0009	0.7316±0.0011	0.7325±0.0006	0.7496±0.0042
5	0.7318±0.0004	0.7286±0.0007	0.7368±0.0005	0.7505±0.0005	NA	0.7496±0.0042
10	0.7254±0.0003	0.7339±0.0005	0.7370±0.0004	0.7479±0.0003	NA	0.7496±0.0042
50	0.7243±0.0004	0.7366±0.0004	0.7378±0.0005	0.7479±0.0004	NA	0.7496±0.0042
100	0.7244±0.0005	0.7352±0.0006	0.7361±0.0005	0.7496±0.0003	NA	0.7496±0.0042
ALL	0.7244±0.0004	0.7377±0.0006	0.7371±0.0005	0.7481±0.0004	NA	0.7496±0.0042

The number of negative examples is varied from the same number of positive examples to the number of all possible negative examples. NA means that it was not computationally feasible.

### 4.3 Biological interpretation of extracted drug substructures and protein domains

We examined the extracted drug substructures and protein domains from biological viewpoints. Because of space limitation, we discuss a few examples below by decreasing order in the weights in the case of the L1LOG algorithm.

The first feature corresponds to PF00106 (short chain dehydrogenase) and SUB158 (three or more ring of size 5). This substructure is present in 34 molecules of the DrugBank, and in particular in the NADH molecule (DB00157). Molecules of the NADH/NAD+ couple are coenzymes found in all living cells and are involved in redox reactions. Enzymes catalyzing oxidation reactions use NAD+ as an oxidating agent, while enzymes catalyzing reduction reactions use NADH as a reducing agent. The PF0106 domain is found in a large number of NAD-dependent oxydoreductases, such as estradiol 17-beta-deshydrogenase (P14061 at Uniprot) or 15-hydroxyprostaglandin dehydrogenase (P15428 at Uniprot).

The second feature corresponds to PF00255 (glutathione peroxidase) and SUB414 (a C-SH group, i.e. a thiol group). Glutathione is a tripeptide that contains an unusual peptide linkage between the amine group of a cystein and the carboxyl group of a glutamate side chain (gammaGlu-Cys-Gly). The cystein residue of glutathione contains the reductive S-H thiol group, and glutathione is often noted G-SH. G-SH is one of the main antioxidant in living cells, and the redox potential of cell compartments are mainly adjusted by the proportion of its reduced form (G-SH) and its oxidized form (G-S-S-G). Proteins with the glutathione peroxidase domain PF00255 catalyse oxidation of G-SH into G-S-S-G. Therefore, the association of the PF00255 domain to the SUB414 is quite consistent on a biological point of view.

The third feature corresponds to PF01126 (heme oxygenase) and SUB158 (three or more ring of size 5). As in the case of PF00106, proteins containing the heme oxygenase domain are oxydoreductases that catalyse the degradation of heme, using NADH/NAD+ as cofactors. This probably explains the extracted association of PF01126 and SUB158.

The fifth feature corresponds to the association of PF03171 (oxygenase superfamily) and SUB687 (O = C-C-C-C-O). Enzymes with the PF03171 domain are oxydoreductases that play a role in various patways such as lysine degradation or DNA repair. Although their functions might be very different, these enzymes all use iron and vitamine C (DB00126) as cofactors. Vitamine C contains the SUB687 chemical substructure, which might explain this feature.

The eighth feature corresponds to the association of PF00167 (fibroblast growth factor) and SUB409 (S-OH). Proteins with the PF00167 domain bind heparin and play an important role in the regulation of cell survival, division and differentiation. Heparin is a highly sulfated glycosaminoglycan containing several S-OH substructures. This substructure is also present in Pentosan polyphosphate (DB00686), an heparin-like drug with anticoagulant properties, that binds to fibroblast growth factors. DB00686 also contain the S-OH substructure, which explains the corresponding feature.

Taken together, the above analysis shows that the best ranked features are biologically meaningful, which is an interesting result. This shows that the proposed algorithms are able to extract the most significant pairs of protein domains and chemical substructures that drive protein-ligand interactions.

## 5 DISCUSSION AND CONCLUSION

In this article we proposed a novel method to identify the underlying associations between drug chemical substructures and protein domains in drug–target interaction networks, based on sparsity-induced binary classifiers. The originality of the proposed method lies in the use of all known protein–ligand interactions across different protein families and the interpretability of the predictive model.

Both our proposed *L*_1_-regularized classifiers and the previous SCCA method are similar in that they induce sparsity into model weights. Although SCCA introduces a sparsity into each canonical component, many features are going to be extracted in total from multiple canonical components, and the same protein domains tend to appear in many different canonical components. Thus, one can suffer from choosing biologically meaningful features from a large number of extracted features. On the other hand, our method successfully extracted a relatively small number of features, which is beneficial for easier biological interpretation. However, our *L*_1_-regularized classifiers can not completely replace SCCA, because each canonical component of SCCA has its own biological meaning. Such property of SCCA is useful when one wants to associate a set of chemical substructures with a set of protein domains.

The proposed method can be used, as soon as drug molecules and target proteins can be represented by descriptors (chemical substructures and protein domains in this study). However, a limitation of the proposed method is that the performance depends on the definition of chemical substructures of drugs and functional domains of target proteins, so the model cannot generalize to chemical substructures or protein domains absent from the learning set. The use of more complete descriptors such as Daylight and Dragon for drugs and other amino acid sequence properties for proteins (Shen *et al.*, 2007) may improve the generalization properties of the method.

*Funding:* Y.T. is supported by MEXT Kakenhi 20589824, the research fellowship from the Japan Society for the Promotion of Science (JSPS) for Young Scientists.

*Conflict of Interest:* none declared.
